# Perspectives of vector management in the control and elimination of vector-borne zoonoses

**DOI:** 10.3389/fmicb.2023.1135977

**Published:** 2023-03-21

**Authors:** Meng Li Wong, Zulhisham Zulzahrin, Indra Vythilingam, Yee Ling Lau, I-Ching Sam, Mun Yik Fong, Wenn-Chyau Lee

**Affiliations:** ^1^Department of Parasitology, Faculty of Medicine, Universiti Malaya, Kuala Lumpur, Malaysia; ^2^Department of Medical Microbiology, Faculty of Medicine, Universiti Malaya, Kuala Lumpur, Malaysia; ^3^Department of Medical Microbiology, University Malaya Medical Centre (UMMC), Kuala Lumpur, Malaysia; ^4^A*STAR Infectious Diseases Labs (A*STAR ID Labs), Agency for Science, Technology and Research (A*STAR), Singapore, Singapore

**Keywords:** zoonoses, vector-borne, vector management, control, prevention

## Abstract

The complex transmission profiles of vector-borne zoonoses (VZB) and vector-borne infections with animal reservoirs (VBIAR) complicate efforts to break the transmission circuit of these infections. To control and eliminate VZB and VBIAR, insecticide application may not be conducted easily in all circumstances, particularly for infections with sylvatic transmission cycle. As a result, alternative approaches have been considered in the vector management against these infections. In this review, we highlighted differences among the environmental, chemical, and biological control approaches in vector management, from the perspectives of VZB and VBIAR. Concerns and knowledge gaps pertaining to the available control approaches were discussed to better understand the prospects of integrating these vector control approaches to synergistically break the transmission of VZB and VBIAR in humans, in line with the integrated vector management (IVM) developed by the World Health Organization (WHO) since 2004.

## Introduction

Zoonoses are infections transmitted from animals to humans (Murphy, 1998). In fact, “zoonosis” is a relatively new word coined by German scientist Rudolf Virchow in the late 19th century, which combines Greek words “zoon” (animal) and “noson” (disease) (Chomel, 2009). Due to increased overlap of habitats by humans and wildlife, climate change, certain economic, cultural and dietary practices, as well as invasion of alien species and convenient international travels, the healthcare and economic burden exerted by zoonoses has increased significantly (Woolhouse and Gowtage-Sequeria, 2005; Jones et al., 2008; Grace et al., 2012; Karesh et al., 2012; Kulkarni et al., 2015). Zoonoses are caused by a variety of pathogens encompassing viruses, bacteria, parasites, fungi, and prions (Taylor et al., 2001; Woolhouse and Gowtage-Sequeria, 2005; Jones et al., 2008). Theoretically, the transmission of a zoonosis can be prevented by segregating humans and the animals that serve as natural hosts of the pathogen (Chomel, 2009; Karesh et al., 2012). However, control and prevention strategies may face additional challenges when the zoonosis is vector-borne, as reflected by knowlesi malaria, a potentially fatal zoonosis transmitted by simio-anthropophilic anopheline mosquitoes (Tan et al., 2008; Jiram et al., 2012; Vythilingam et al., 2014; Wong et al., 2015; Lau et al., 2016). Similar obstacles happen with vector-borne infections possessing animal reservoirs, such as the tsetse fly-transmitted *Trypanosoma brucei*, the sand fly-transmitted leishmaniasis, and the mosquito-borne Sindbis virus (SINV), Zika virus (ZIKV), and yellow fever virus (Balfour, 1914; Njiokou et al., 2006; Singh et al., 2013; Vorou, 2016; Steyn et al., 2020; Kushwaha et al., 2022). Since the involving animals cannot be culled just to break the transmission circuit to humans (Lee et al., 2022), vector control is a critical component of breaking the transmission of vector-borne zoonoses (VBZ) and vector-borne infections with animal reservoirs (VBIAR). Vector control programs aim at either reducing the population of the vectors, or avoiding, if not reducing the exposure of the targeted vectors to humans (Wilson et al., 2020). Notably, a wide variety of arthropods and arachnids with different biological behaviors have been verified as medically important disease vectors (Table 1). The diverse array of vectors, animal reservoirs, and activities engaged by humans in the vicinity contribute different challenges to the control and elimination of these diseases. Here, we discussed the main vector control strategies in the current scenario, highlighted the strengths, limitations, and concerns arising from these approaches, knowledge gaps that deserve to be filled, and possibility of integrating multiple approaches of vector management into the control and elimination of VBZ and VBIAR.

**Table 1:** Vector-borne diseases (VBDs) and the respective vectors and animal reservoirs.

VBDs	Causative agent	Vector	Animal reservoir	Refs
Malaria	Human: *Plasmodium falciparum, Plasmodium vivax, Plasmodium malariae, Plasmodium ovale* spp.	Mosquito: *Anopheles* spp.	N/A	Warren and Wharton (1963), Vythilingam et al. (2001, 2005, 2006), da Rocha et al. (2008), Tan et al. (2008), Sinka et al. (2010), Sinka et al. (2011), Jiram et al. (2012), Sinka et al. (2012), Vythilingam (2012), Vythilingam and Hii (2013), Vythilingam et al. (2013), Vythilingam et al. (2014), Wong et al. (2015), Lau et al. (2016), Liew et al. (2021), and Vythilingam et al. (2021)
	Zoonotic: *Plasmodium knowlesi, Plasmodium cynomolgi, Plasmodium inui, Plasmodium simium, Plasmodium brasilianum*	Mosquito: *Leucosphyrus* group of mosquitoes	Simian primates	
Babesiosis	*Babesia microti, Babesia divergens, Babesia duncani, Babesia venatorum*	Tick: *Ixodes* spp.	Cattle, roe deer and rodents	Donnelly and Peirce (1975), Spielman (1976), Lewis and Young (1980), Walter and Weber (1981), Mylonakis (2001), Gray et al. (2002), and Bonnet et al. (2009)
Dengue	Dengue virus (DENV)	Mosquito: *Aedes aegypti, Ae. albopictus, Ae. polynesiensis, Ae. scutellaris* group	Monkey (sylvatic dengue strains)	Siler et al. (1926), Chan et al. (1971), Rosen and Gubler (1974), Gubler (1988), Moore and Mitchell (1997), Rigau-Perez and Gubler (1997), Scott et al. (1997), Hotta (1998), Tsuda et al. (2002), Gubler et al. (2007), Lambrechts et al. (2010), and Higa (2011)
Yellow fever	Yellow Fever virus (YFV)	Mosquito: *Aedes* spp.*, Haemagogus* spp.	Monkeys	Haddow (1969), Barrett and Higgs (2007), and Young et al. (2014)
Chikungunya	Chikungunya virus (CHIKV)	Mosquito: *Ae. aegypti*, *Ae. albopictus*	Primates	Niyas et al. (2010) and Kumar et al. (2012)
O’nyong’nyong fever	O’nyong’nyong virus (ONNV)	Mosquito: *Anopheles* spp.	N/A	Shore (1961), Johnson (1988), and Young et al. (2014)
Sindbis fever	Sindbis virus (SINV)	Mosquito: *Culex* spp.*, Culiseta* spp.	Birds	Laine et al. (2004), Young et al. (2014), and Sang et al. (2017)
Zika	Zika virus (ZIKV)	Mosquito: *Aedes* spp.	Primates	Dick (1952), Marchette et al. (1969), and Vorou (2016)
Rift Valley fever	Rift Valley fever virus (RVFV)	Mosquito: *Aedes* spp.*, Culex* spp.	Ruminants	Davies (1975), Davies and Highton (1980), Chevalier et al. (2004), and Sang et al. (2017)
West Nile fever	West Nile virus (WNV)	Mosquito: *Culex* spp.	Birds	Taylor and Hurlbut (1953) and Shirato et al. (2005)
Japanese encephalitis	Japanese encephalitis virus (JEV)	Mosquito: *Culex* spp., *Ae. togoi, Ae. japonicus, Ae. vexans nipponii, An. annularis, An. vagus*	Birds, pigs (amplifier host)	Sucharit et al. (1989), Vythilingam et al. (1994), Vythilingam et al. (1995), Vythilingam et al. (1997), Weng et al. (1999), Das et al. (2005), Nitatpattana et al. (2005), van den Hurk et al. (2006), Seo et al. (2013), and de Wispelaere et al. (2017)
Murray Valley encephalitis	Murray Valley encephalitis virus (MVEV)	Mosquito: *Culex annulirostris*	Birds	Marshall (1988), Mackenzie et al. (1994), Kurucz et al. (2005), and Floridis et al. (2018)
Tick-borne encephalitis	Tick-borne encephalitis virus (TBEV)	Tick: *Ixodes* spp., *Dermacentor* spp.	Small mammals	Kozuch and Nosek (1971), Labuda and Randolph (1999), and Biernat et al. (2014)
Kunjin encephalitis	Kunjin virus (KUNV)	Mosquito: *Culex annulirostris*	Birds	Doherty et al. (1963), Kay et al. (1984), Marshall (1988), Hall et al. (2002), and Hall et al. (2006)
Colorado tick fever	Colorado tick fever virus (CTFV)	Tick: *Dermacentor andersoni*	Squirrels, chipmunks, mice	Florio et al. (1944), Florio and Miller (1948), Florio et al. (1950), and Emmons (1988)
Lymphatic filariasis	Human: *Wuchereria bancrofti, Brugia malayi, Brugia timori*	Mosquito: *Anopheles* spp.*, Culex* spp.*, Aedes* spp.*, Mansonia* spp.	Cats, dogs, monkeys, pangolins (*B. malayi*)	Edeson and Wilson (1964), Cheong et al. (1981), Chiang et al. (1984), Hii et al. (1984), Zahedi and White (1994), Kanjanopas et al. (2001), Vythilingam (2012), Muslim et al. (2013), Vythilingam et al. (2013), WHO (2013), Aagaard et al. (2015), Mulyaningsih et al. (2019), and Nunthanid et al. (2020)
	Zoonotic: *Brugia pahangi*	Mosquito: *Armigeres subalbatus*	Cats and dogs	
Serous cavity filariasis	*Mansonella perstans, Mansonella ozzardi*	Midge: *Culicoides* spp.	N/A	Manson (1891)
Subcutaneous filariasis	Loiasis: *Loa loa*	Deer fly: *Chrysops* spp.	N/A	Kleine (1915), Connal (1921), Macfie and Corson (1922), Fischer et al. (1997), Lawrence (2004), Boussinesq (2006), Kelly-Hope et al. (2017), and Hendy et al. (2018)
	*Mansonella streptocerca*	Midge: *Culicoides* spp.	N/A	
	Onchocerciasis / river blindness: *Onchocerca volvulus*	Black fly: *Simulium* spp.	N/A	
Sleeping sickness (African trypanosomiasis)	*Trypanosoma brucei rhodesiense, Trypanosoma brucei gambiense*	Tsetse fly: *Glossina* spp.	Cattle (*T. brucei rhodesiense*) Primates & ungulates (*T. brucei gambiense*)^#^	Bruce (1895, 1915) and Njiokou et al. (2006)
Chagas disease (American trypanosomiasis)	*Trypanosoma cruzi*	True bug/kissing bug/triatomine/reduviid bug: *Rhodnius prolixus, Triatoma infestans*	Small rodents	Chagas (1909), Jurberg and Galvão (2006), Rassi et al. (2010), and WHO (2015)
Leishmaniasis	*Leishmania*	Phlebotomine sandfly: *Phlebotomus* spp.*, Lutzomyia* spp.	Dogs	Swaminath et al. (1942), Mukhopadhyay et al. (2000), and Guerbouj et al. (2007)
Epidemic typhus (louse-borne typhus)	*Rickettsia prowazekii*	Human body louse: *Pediculus humanus humanus*	Flying squirrels (sylvatic typhus)	McDade et al. (1980), McDade and Newhouse (1986), and Durden (2019)
Rocky Mountain spotted fever (RMSF)	*Rickettsia rickettsii*	Tick: *Dermacentor variabilis, Dermacentor andersoni, Rhipicephalus sanguine*	Small mammals	Kohls (1947) and Ahantarig et al. (2013)
Queensland tick typhus (QTT)	*Rickettsia australis*	Tick: *Ixodes holocyclus*, *I. tasmania*	Bandicoots, rodents	Fenner (1946), Domrow and Derrick (1964), Sexton et al. (1991), and Barker and Walker (2014)
Scrub typhus	*Orientia tsutsugamushi*	Mite: *Leptotrombidium* spp.	Rodents	Shirai et al. (1981), Pham et al. (2001), Lerdthusnee et al. (2003), and Weitzel et al. (2022)
Tularemia (rabbit fever)	*Francisella tularensis*	Tick: *Amblyomma* spp.*, Dermacentor* spp.*, Haemaphysalis* spp.*, Ixodes* spp.	Rabbits, hares, other small rodents	Parker et al. (1924), Gurycová (1998), Sjöstedt (2007), Kugeler et al. (2009), Männikkö (2011), Maurin et al. (2011), Yeni et al. (2021), and Troha et al. (2022)
		Deer fly: *Chrysops discalis*	Deers	
Lyme disease	*Borrelia burgdorferi, Borrelia mayonii*	Tick: *Ixodes* spp.	Avians, mammals	Wilson et al. (1985), Steere (2001), Lo Re et al. (2004), and Couper et al. (2020)
Bubonic plague	*Yersinia pestis*	Oriental rat flea: *Xenopsylla cheopis*	Rodents	Bacot and Martin (1914), Bacot (1915), Burroughs (1947), and Pollitzer (1954)
Anaplasmosis	*Anaplasma phagocytophilum*	Tick: *Ixodes* spp.	Mammals, birds	Chen et al. (1994), Ohashi et al. (2005), Katargina et al. (2012), and Bakken and Dumler (2015)
Ehrlichiosis	*Ehrlichia chaffeensis, Ehrlichia ewingii, Erhlichia muris eauclairensis*	Ticks: *Amblyomma* spp., *Ixodes* spp.	Mammals	Anderson et al. (1993), Lockhart et al. (1997), and Ganguly and Mukhopadhayay (2008)

# Humans are the main reservoir for *T. brucei gambiense* but this parasite has been isolated from primates and ungulates.

## The trilogy of vector control strategies

In general, vector control strategies can be classified into chemical, biological and environmental management approaches (Bos, 1991). Each of these approaches gained research and public attention at different time points and comes with its own advantages and disadvantages. These approaches are inter-related, where simultaneous application of multiple approaches can produce either synergistic effect against the propagation of vectors, or antagonistic effect that disputes the vector control program. Therefore, a thorough understanding on each vector control approach is crucial for a successful vector control that can lead to the eradication of respective vector-borne diseases (WHO, 2012). This is particularly crucial for the management of VBZ and VBIAR, as the transmission profiles of these infections are usually more complex, involving more organisms. In fact, some of these diseases have multiple transmission cycles. For example, *Trypanosoma cruzi* has an urban transmission cycle involving humans, and sylvatic cycle involving wildlife (Orozco et al., 2013), whereas yellow fever virus has sylvatic, intermediate/savannah and urban transmission cycles (Valentine et al., 2019; Cunha et al., 2020; Gabiane et al., 2022). Of note, each transmission cycle may involve different vectors with distinct biological properties and behaviors that further complicate transmission blocking *via* vector control program. Worse still, many of these infections have incompletely deciphered transmission risk factors (Swei et al., 2020). Due to such complexity, a well-designed multi-pronged strategy that integrates multiple approaches may be more suitable to control the transmission of VBZ and VBIAR.

## Environment management approach

**Figure 1:**
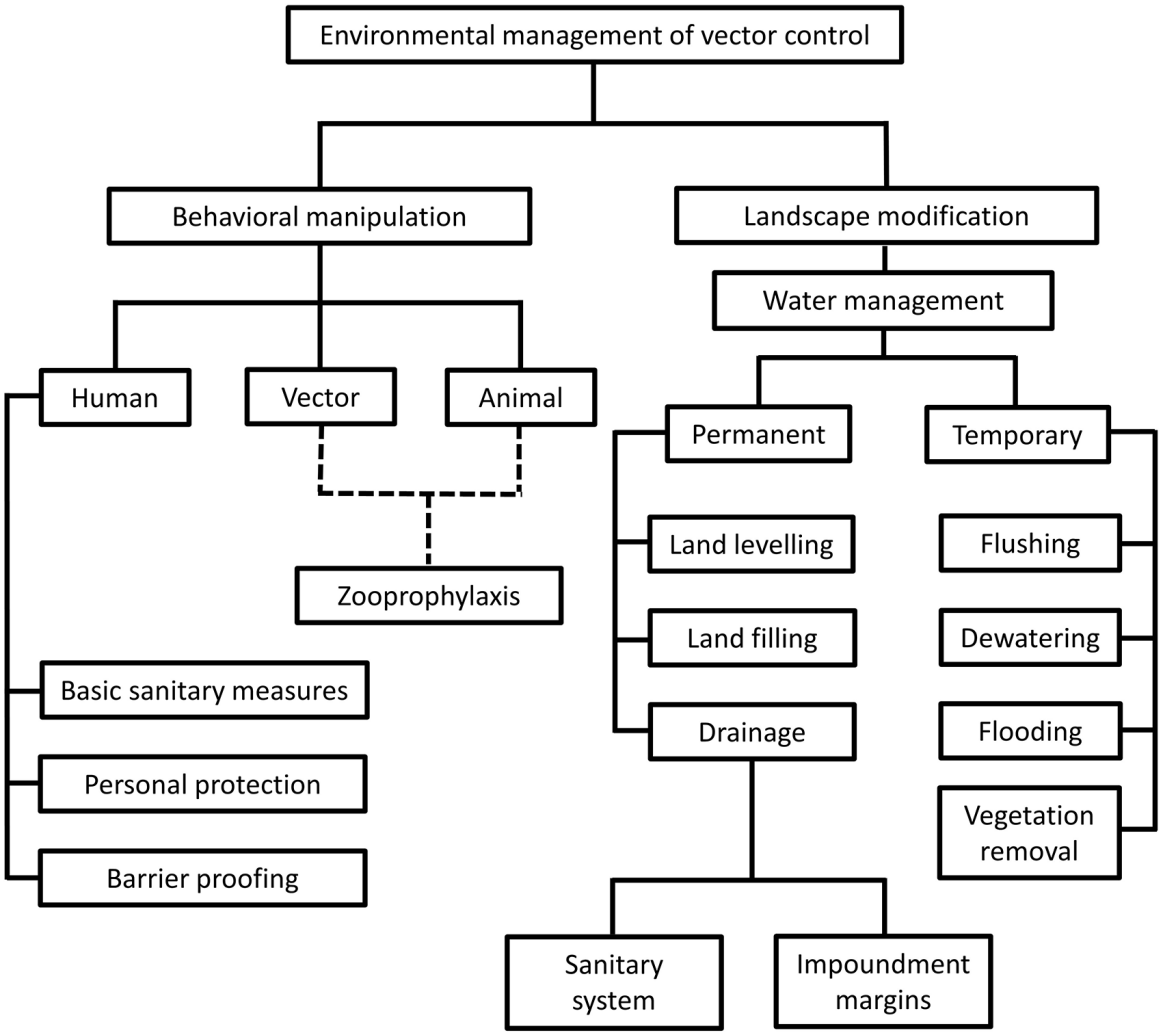
Different strategies under the environmental management of vector control. This approach revolves around behavioral alteration of humans, animals and vectors, as well as landscape modification, to create barriers between humans and vectors. Of note, the “zooprophylaxis” under “behavioral manipulation” involves the introduction of animals that are not pathogen reservoirs, to distract the blood-seeking vectors from humans and animals that serve as natural reservoirs of pathogens. This method involves behavioral alteration of animals and vectors, as indicated by the dotted lines in the diagram. On the other hand, landscape modification consists of permanent and temporary water management strategies to change the breeding environment of vectors.

The environment management approach was the predominant vector control method prior to World War II (WWII). During this period, comprehensive understanding on local vector behavior and ecology dynamics, along with specifically tailored environmental management plans were the prerequisites toward a successful vector control (Quiroz-Martinez and Rodriguez-Castro, 2007; Wilson et al., 2020). The environment approach revolves around behavioral manipulation and landscape modification (Figure 1). Behavioral manipulation can be directed at humans, animals or the vectors involved (Ault, 1994). For example, community members can be trained to practice good sanitary measures around their housing compound, set up barrier proofing against mosquitoes (such as usage of bed net and mosquito screens), and employs personal protection when exploring places with high vector density (Demers et al., 2018; Wilson et al., 2020). Zooprophylaxis can be employed to distract vectors from biting humans (or animals that serve as natural reservoirs of the targeted pathogen), by introducing another animal with similar or better feeding attractiveness to the targeted vectors (Charlwood et al., 1985; Sousa et al., 2001). In this context, the mosquito behavior is manipulated. On the other hand, landscape modification revolves around temporary and permanent strategies of water management, with the goal of removing suitable breeding grounds for the vectors (Watsons, 1921). Vector control *via* environment management has been employed against the transmission of malaria (Le Prince and Orenstein, 1916; Watsons, 1921; Utzinger et al., 2001; Lindsay et al., 2002; Ferroni et al., 2012), lymphatic filariasis (van den Berg et al., 2013; Davis et al., 2021), yellow fever (Le Prince and Orenstein, 1916; Soper and Wilson, 1943), African trypanosomiasis (Jackson, 1941; Jackson, 1943; Jackson, 1948; Scott, 1966; Hargrove, 2003; Headrick, 2014), and leishmaniasis (Busvine, 1993; Steverding, 2017) in different parts of the world. However, this approach does not work in a “one size fits all” manner. For example, the zooprophylaxis approach reported promising results in Papua New Guinea and São Tomé (Charlwood et al., 1985; Sousa et al., 2001). However, this approach experienced failure in places such as Ethiopia, the Gambia, and Pakistan (Bouma and Rowland, 1995; Ghebreyesus et al., 2000; Bøgh et al., 2001). Such contradicting outcomes were due to various factors, including the types of vectors targeted in these studies. Indeed, the success of zooprophylaxis relies on the prerequisites that the involving vectors must be zoophilic and exophilic (outdoor feeders), in addition to the adequate segregation between the human and animal living spaces (Asale et al., 2017).

A thorough evaluation and understanding on the stakeholders and targeted areas, along with long-term engagement (commitment) by the government and community members are needed to ensure a higher success rate of vector control *via* environment management. However, these can only be achieved with adequate time, financial support, and sustainable manpower. In addition, the benefits brought by this approach may be shadowed by unpredictable and potentially irreversible negative impact cast upon the environment, as exemplified by the bush clearing effort in parts of Africa during the 1950s and 1960s to control the population of tsetse flies (Scott, 1966; Hargrove, 2003; Pilossof, 2016). Hence, this vector control approach may not be an ideal solution for all diseases. Nevertheless, this approach is still a valuable tool for a sustained elimination of the targeted vector-borne diseases, provided that the approach is designed carefully by taking all biological, environmental, legal and socio-economic factors into consideration.

## Chemical vector control

Chemical vector control strategies have gained popularity, especially after the WWII, due to the rapid and potent effect of these methods. The development, marketing and application of various insecticides has been the mainstream of chemical vector control strategy. Attempts to employ chemicals for pest control were recorded as early as the 1840s (Table 2). However, the discovery of dichloro-diphenyl-trichloroethane (DDT) revolutionized the approach to control vector population. The insecticidal properties of DDT were discovered in 1939 (Mellanby, 1992; Davies et al., 2007). Following the halted supply of chrysanthemum-derived pyrethrum from Japan due to WWII, DDT became the mainstream chemical player in vector control (Wilson et al., 2020), especially after its involvement in the successful control of typhus outbreak in Europe (Wheeler, 1946). Following this much publicized success against lice, DDT was proven to be potent against many other vectors such as the mosquito, tsetse fly, sandfly and blackfly (Ismail et al., 1975; Loyola et al., 1990, 1991; Roberts and Alecrim, 1991; Casas et al., 1998; Hargrove, 2003; Dias, 2007; Rijal et al., 2019). Nevertheless, the negative impacts brought by DDT to non-targeted organisms and environment were discovered after years of mass application. As a result, the application of this powerful chemical was discontinued abruptly in the 1970s (Davies et al., 2007). Subsequently, other insecticide groups such as organophosphates, carbamates and synthetic pyrethroid gained popularity in many vector control programs. This has stimulated various chemical-oriented vector combating strategies, such as the long-lasting insecticidal net (LLIN), indoor residual spraying (IRS), as well as outdoor residual spraying (ORS; Bonsall and Goose, 1986; Bhatt et al., 2015; Rohani et al., 2020; Tangena et al., 2020; Chaumeau et al., 2022).

**Table 2:** Brief overview of insecticides in vector control.

Year	Description	Methods	Refs
1840	Discovery of insecticidal properties of a *Tanacetum* (*Chrysanthemum*) *cinerariifolium* (Compositae)-derived compound (pyrethrum), subsequently its successful extraction and commercial production	N/A	Ujváry (2010)
1930s	Discovery of insecticidal properties of organophosphates (OP) and carbamate.	N/A	Hill (1995) and Glaser (1999)
1939	Discovery of insecticidal properties of dichlorodiphenyltrichloroethane (DDT) against flies, mosquitoes and beetles by Paul Muller	N/A	Mellanby (1992) and Davies et al. (2007)
1943	First application of DDT in Italy to control typhus epidemic	Dusting 10% DDT powder onto clothing of infested individuals to kill body lice	Wheeler (1946)
1946–1991	Widespread application of DDT and other organochlorines (OC) in various locations to control vector-borne diseases	Aerial spraying and indoor residual spraying (IRS)	Ismail et al. (1975), Loyola et al. (1990), Loyola et al. (1991), Roberts and Alecrim (1991), Casas et al. (1998), and Hargrove (2003)
1949	Development of the first synthethic pyrethroids	N/A	Davies et al. (2007) and Matsuo (2019)
1955–1969	Introduction and implementation of Global Malaria Eradication Program by WHO	Control program varied across different locations	Najera et al. (2011)
1972	DDT usage was banned by US Environment Agency	N/A	Mellanby (1992) and Davies et al. (2007)
1970s – present	Development of pyrethroid-treated net (ITN) for malaria control. Organophosphates and carbamates are more widely used as replacements for OC due to hazardous effect imposed by DDT	Organophosphates: residual spraying, space spraying and larviciding. Carbamates: residual spraying	Bonsall and Goose (1986), Dorta et al. (1993), van den Berg et al. (2012), Tangena et al. (2020), and van den Berg et al. (2021a,b)

Various chemicals have been developed and marketed as readily available larvicides and adulticides. The high availability and instantaneous killing effect of these products have created a dogma that the chemicals are the best way forward in vector management (Casida and Quistad, 1998; Thomas, 2018). Nevertheless, the biology of arthropods plays a critical role in determining the success rate of insecticide-mediated vector control programs. For instance, IRS and LLIN are not suitable for exophagic and exophilic mosquitoes with peak biting time in the early evening (Dolan et al., 1993; Rohani et al., 1999; Smithuis et al., 2013; Wong et al., 2015). Besides, behavioral adaptation of endophilic mosquitoes toward avoidance of insecticide-treated houses or rapid exit from the insecticide-treated buildings will minimize the exposure of these vectors to the insecticides, compromising the efficacy of the applied insecticide (Killeen, 2014). Importantly, the rampant usage of these chemicals has fuelled insecticide resistance in arthropods (Kleinschmidt et al., 2018; Tangena et al., 2020). Moreover, these chemicals may cast negative impacts to the ecosystem, although of lower toxicity than DDT. For example, synthetic pyrethroids are harmful to aquatic environment (Thatheyus and Selvam, 2013; Prusty et al., 2015), whereas organophosphates poisoning remains prevalent among communities involved in agricultural industry, despite being classified as non-persistent pesticides (Jaipieam et al., 2009; Kaushal et al., 2021). Due to these disadvantages, the chemical approach must be considered carefully in vector control programs against VBZ and VBIAR, particularly those with sylvatic transmission cycle.

Despite the non-specific harm to the environment due to their toxicity, the rapid and potent effect of insecticides against different vectors grants them the high popularity in pest and vector control. Many researchers have investigated ways of accelerating the degradation of these chemicals to minimize their adverse effects to the environment, while retaining their potency against the pests (Zhang and Qiao, 2002; Kaushal et al., 2021; Zhao et al., 2022). Meanwhile, the discovery of pyrethrum from chrysanthemum plant continues to inspire scientists to find novel compounds that can serve as bio-insecticides. For example, bioactive metabolites of *Streptomyces* have been reported to demonstrate good potential of becoming bio-insecticide candidates (Amelia-Yap et al., 2022). Such discovery has been driven by the need of novel, environment-friendly insecticide compounds, following rapid development of insecticide resistance and concerns over environment harm cast by chemical-based insecticides.

## Vector biocontrol approach

**Figure 2:**
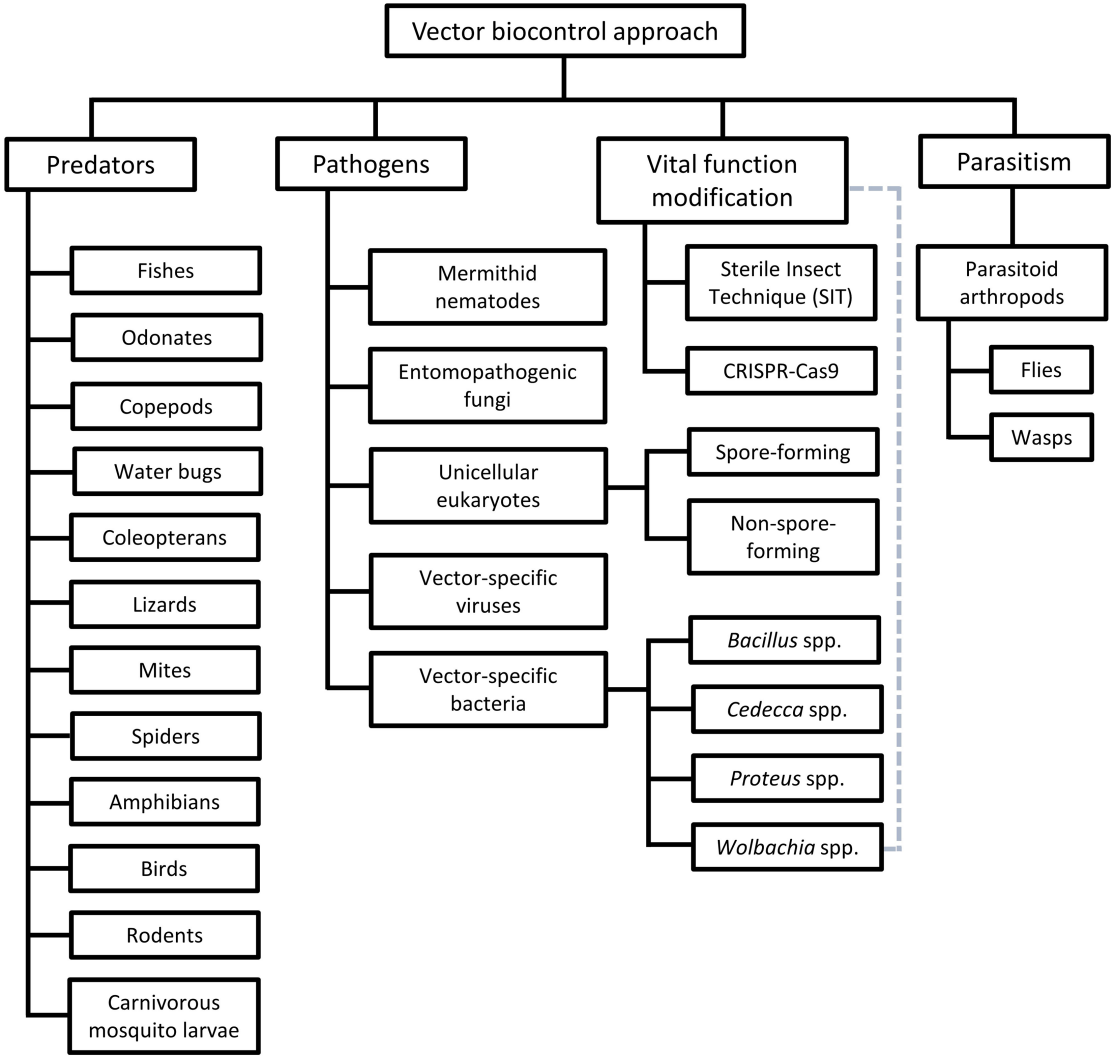
Different groups of vector biocontrol approach. Gray dotted lines reflect the characteristics of the *Wolbachia* method that combines the features of biocontrol approaches mediated by pathogens and vital function modification.

Among the vector control strategies, biocontrol approaches have received increasing attention and popularity over the past two decades. Therefore, various organisms and strategies have been put forward as potential vector biocontrol candidates. In general, biocontrol approach explores the potential of using organisms and microorganisms to control the vector population (van den Bosch et al., 1982; Kamareddine, 2012; Okamoto and Amarasekare, 2012; Benelli et al., 2016; Huang et al., 2017; Kwenti, 2017; Thomas, 2018), based on the natural predation, pathobiological or parasitism relationship between the candidates and the targeted vectors (Table 3). Biological manipulation targeting certain vital functions of the vectors have been explored as a new approach in vector biocontrol (Gillette, 1988; Iturbe-Ormaetxe et al., 2011; Benelli et al., 2016). Theoretically, the biocontrol approach is more target-specific, thus of lower risk of imparting off-target effects to the environment. Prior to the new millennium, biocontrol approach was not as widely applied as its chemical and environmental counterparts, due to the relative ease of implementing the other two approaches (Quiroz-Martinez and Rodriguez-Castro, 2007; Shaalan and Canyon, 2009; Vershini and Kanagappan, 2014; Vinogradov et al., 2022). Nevertheless, biocontrol approach has received increasing attention following encouraging results obtained from the mass-application of *Wolbachia*-infected *Aedes aegypti*, genetically modified mosquitoes, sterile male triatomine bugs and tsetse flies. In fact, with the increased prevalence of VBZ and VBIAR, vector biocontrol approach may offer novel and sustainable strategies to control the transmission of these infections. Biological control approach can be categorized based on the natural relationship between the biocontrol agents and the respective vectors (Figure 2), as elaborated in the next few paragraphs of this review.

**Table 3:** List of available vector biocontrol agents.

Biocontrol agent type	Biocontrol agent	Commonly used strains/species	Remark	Limitation	Refs
Predator	Larvivorous fish	*Aphanius dispar Aplocheilus* spp. *Chanda nama Colisa* spp. *Carassius auratus Catla catla Cirrhinus mrigala Ctenopharyngodon idella Cyprinodontidae Cyprinus carpio Danio rerio Gambusia affinis Labeo rohita Macropodus cupanus Nothobranchius guentheri Oreochromis* spp. *Oryzias melastigma Poecilia reticulata Sarotherodon niloticus Tilapia* spp.	Natural predator of larvae: reduces number of mosquito larvae	A threat to native aquatic fauna. Inconsistency in terms of efficacy	Connor (1922), Menon and Rajagopalan (1978), Rupp (1996), Walton (2007), Chandra et al. (2008a), Louca et al. (2009), Griffin and Knight (2012), and Subramaniam et al. (2015)
	Dragonfly	Nymph and adult *Anax immaculifrons Brachydiplax sobrina Neurothemis fluctuans Orthetrum chrysis Orthethrum sabina*	Reduces the number of the vector population through feeding on immature and adult	Critically affected by water quality, thus field application can be limited	Sebastian et al. (1990), Singh et al. (2003), Chatterjee et al. (2007), Quiroz-Martinez and Rodriguez-Castro (2007), Shaalan and Canyon (2009), Vershini and Kanagappan (2014), Vatandoost (2021), and Ramlee et al. (2022)
	Larvivorous mosquito larva	*Psorophora* subgenus *Psorophora Sabethes cyaneus Toxorhynchites* spp. *Lutzia* spp. *Sabethes* spp. *Trichoprosopon* spp. *Runchyomyia* spp. *Culex fuscanus Anopheles barberi Tripteroides* spp. *Topomyia* spp. *Wyeomyia* subgenus *Dendromyia Eretmapodites* spp. *Aedes* subgenus *Alanstomea Aedes* subgenus *Mucidus*	Decreases number of mosquito larvae	Spatial limitations for application, especially for some sylvatic species. Risk of cannibalism among larvivorous mosquito larva	Chapman (1974), Lounibos (1980), Focks et al. (1985), Annis et al. (1989), Annis et al. (1990), Rawlins et al. (1991), Brown (1996), Mogi and Chan (1996), Amalraj and Das (1998), Collins and Blackwell (2000), Aditya et al. (2006), Benelli et al. (2016), Huang et al. (2017), Donald et al. (2020), and Hancock et al. (2022)
	Larvivorous copepod	*Megacyclops* spp. *Mesocyclops* spp. *Macrocylops* spp.	Reduces mosquito larvae density	Copepods are affected by water temperature, low oxygen content and accumulation of toxins in water. Some copepods are intermediate host for guinea-worm and fish tape worm	Marten et al. (1989), Lardeux et al. (1992), Manrique-Saide et al. (1998), Schaper (1999), Vu et al. (2005), Marten and Reid (2007), Soumare and Cilek (2011), Mahesh Kumar et al. (2012), and Vinogradov et al. (2022)
	Beetle	Diving beetle (Dystiscidae) Water scavenger beetle (Hydrophilidae)	Reduces number of vector immatures	Incomplete habitats overlap. Alternative prey preference. Emigration. Limited research	Juliano and Lawton (1990), Lundkvist et al. (2003), Chandra et al. (2008b), Shaalan and Canyon (2009), and Vinogradov et al. (2022)
	Water bug	Backswimmer (Notonectidae) Giant water bugs (Belostomatidae) Waterboatmen (Corixidae)	Reduces number of vectors: feeds by holding its prey with pincers and injecting a strong liquefying enzyme into it	Greatly affected by water quality, limiting its spatial reach to the vectors. Difficulty in mass production	Bay (1974), Murdock et al. (1984), Venkatesan and Jeyachandra (1985), Sankaralingam and Venkatesan (1989), Aditya et al. (2004), Aditya et al. (2005), Shaalan et al. (2007), Shaalan and Canyon (2009), Selvarajan and Kakkassery (2019), and Vinogradov et al. (2022)
	Mite	*Acari* spp. *Eustigmaeus johnstoni* (affects sand fly) *Pimeliaphilus plumifer* (affects true bugs)	Feeds on vector immature. Affects the physiological aspects of vector: reduces nymph molting rate, reduces adult longevity, increases mortality in 3rd–5th instar nymph, reduces number of viable eggs laid by infected female	Difficulty is mass-rearing	Martinez-Sanchez et al. (2007), Badakhshan et al. (2013), and Dinesh et al. (2014)
	Spider	Web-building spider. Hunting spiders (Active and passive hunter)	Feeds on vector immatures and adults	Consideration on different biological factors to ensure successful establishment of control	Ximena et al. (2005), Hadole and Vankhede (2013), Fischhoff et al. (2018), and Ndava et al. (2018)
	Lizard	*Gehydra dubia Hemidactylus frenatus Tarentola mautitanica* (prey: true bug)	Feeds on adults	Possible threat to native fauna	Castello and Gil Rivas (1980) and Canyon and Hii (1997)
	Frog and toad	*Bufo* spp. *Euphlycytis* spp. *Hoplobatrachus* spp. *Polypedates cruciger Ramanella* spp.	Predates on eggs of mosquito	Can be invasive toward native fauna	Raghavendra et al. (2008) and Bowatte et al. (2013)
	Bird	Scrub jay Chicken Yellow-billed oxpecker (*Buphagus africanus*) Red-billed oxpecker (*Buphagus erythrorhycus*)	Predates on ticks (scrub jay: ticks on deer; chicken: ticks on cattle; yellow-billed oxpecker: ticks on buffaloes; red-billed oxpecker: ticks on ungulate)	Oxpecker could induce wound enlargement on the mammalian host given that it prefers host with most ticks. Assessment of tick population needs to be performed before introduction programme (scrub jay and oxpeckers)	Moreau (1933), van Someren (1951), Mundy and Cook (1975), Bezuidenhout and Stutterheim (1980), Isenhart and DeSante (1985), Hassan et al. (1991), Mooring and Mundy (1996), Weeks (1999), and Plantan et al. (2012)
	Rodent	*Sorex araneus*	Predates on ticks	Not advisable as rodent transmits several diseases	Short and Norval (1982)
Parasitism	Parasitoid arthropods	Tachinid fly (parasitizes true bug) Chalcid wasp (parasitizes tick) *Ixodiphagus hookeri* (Encyrtid wasp-parasitizes tick)	Immatures of vector is attacked when the eggs of the parasitoid arthropods hatch and feed on it	Highly sensitive to insecticides. Mass-rearing in laboratory can be difficult, especially the diet preparation	Mather et al. (1987), Tijsse-Klasen et al. (2011), Wang et al. (2014), Kwenti (2017), Wang et al. (2019), and Buczek et al. (2021)
Pathogens	Nematode	Mermithid nematode (*Perutilimermis culicis*, *Romanomermis* spp., *Reeseimermis nielseni*, *Diximermis peterseni*, *Hydromermis churchillensis*). Rhabditoid nematode (*Neoaplectana carpocapsae*) Stenernematid nematode (ticks)	Parasitic relationship: Reduces number of mosquitoes. Causes biological castrations through interference in mosquito reproduction	Limited resources on the parasitic effects of nematodes against the adult mosquitoes. Environmental parameters limitations such as temperature, pH, salinity, and oxygen level	Petersen et al. (1972), Petersen and Willis (1972), Reynolds (1972), Chapman (1974), Mitchell et al. (1974), Levy and Miller (1977), Molloy and Jamnback (1977), Zhioua et al. (1995), Peng et al. (1998), Samish and Glazer (2001), Secundio et al. (2002), and Poinar (2018)
	Entomopathogenic fungus	*Beauveria* spp. *Coelomomyces* spp. *Culicinomyces* spp. *Entomophthora* spp. *Lagenidium* spp. *Metarhizium* spp. *Phytium* spp. *Smittium* spp. *Fusarium oxysporum*	Upon contact to external cuticle, toxins are released by the infective spores. Modifies physiology of insect: reduces likelihood for blood-feeding, survival, and fecundity	Slow killing. Production of zoospore is difficult and affected by UV irradiation. Some strains can affect non-target arthropods. *Beauveria bassiana* are inactive against adults in laboratory (*Anopheles, Aedes, Culex*). *Entomophthora coronata* has been reported to cause phycomycosis in man and horses. *Smittium* spp. has reduced pathogenicity against mosquitoes	Clark et al. (1966), Clark et al. (1968), Anderson and Ringo (1969), Ginsberg et al. (2002), Scholte et al. (2004), Scholte et al. (2007), Paula et al. (2011a,b), and Fischhoff et al. (2018)
	Non-spore-forming unicellular eukaryotes	Ciliate: *Tetrahymena* spp. Flagellate: (*Crithidia* spp. *Blastocrithidia* spp. *Eugregarine Ascogregarina culicis Psychodiella* spp. (found only in sand flies) Schizogregarine: *Caulleryella* spp. Helicosporida)	Stunts growth of larvae and increased mortality. Effects on host’s biological aspects especially on females are more profound in nutrient-deficient conditions	Pathogenicity highly depends on internal and external conditions. Host-specific	Corliss (1954, 1960), Chapman et al. (1967), Anderson (1968), Barrett (1968), McCray et al. (1970), Reynolds (1972), Wu and Tesh (1989), Sulaiman (1992), Mourya et al. (2003), Albicócco and Vezzani (2009), Lantova et al. (2011), and Lantova and Volf (2012, 2014)
	*Microsporida*	*Thelohania* spp. *Nosema* spp. *Pleistophora* spp. *Stempellia* spp.	Swollen thorax and abdomen/ benign subcutaneous pale spots on mosquito larvae. Reduces life span of infected female mosquito	Most of them cannot be transmitted perorally. Spores from different species are difficult to identify morphologically	
	Bacteria	*Bacillus sphaericus Bacillus thuriengiensis Bacillus thuringiensin* var. *thuringiensin Cedecca lapegei Proteus mirabilis* Different Wolbachia strains	Pathobiological effect against vectors: target is killed by an enterotoxin from crystal protein of spore. Suppresses late instars and pupae. Affects reproductive system. Shortens vectors’ life	Inconsistent efficacy	Lacey and Inman (1985), Novak et al. (1986), Arredondo-Jimenez et al. (1990), Hassanain et al. (1997), Robert et al. (1997), Stouthamer et al. (1999), Armengol et al. (2006), Lacey (2007), Panteleev et al. (2007), Hedges et al. (2008), Werren et al. (2008), Brelsfoard and Dobson (2009), Kambris et al. (2009), Moreira et al. (2009a), Wiwatanaratanabutr and Kittayapong (2009), Bian et al. (2010), Ritchie et al. (2010), Ahantarig and Kittayapong (2011), Hoffmann et al. (2011), Iturbe-Ormaetxe et al. (2011), Walker et al. (2011), Mousson et al. (2012), van den Hurk et al. (2012), Bian et al. (2013), Aliota et al. (2016a,b), Dutra et al. (2016), Jeffries and Walker (2016), Ahmad et al. (2017), Chouin-Carneiro et al. (2019) and Nazni et al. (2019)
	Virus	Mosquito-specific densovirus (MDV) Cytoplasmic polyhedrosis virus (CPV or near reovirus) Nuclear polyhedrosis virus (NPV or baculovirus) Deltabaculovirus (dipteran-specific NPVs) *Mosquito Iridescent Virus* (MIV or iridovirus) Entomopoxvirus (EPV)	Intranuclear protein inclusions in *Anopheles subpictus.* Infections in nuclei in midgut and gastric caeca of *An. sollicitans.* Kills fourth instar mosquito larvae	Host-specific. Slow killing, hence, studies are being performed by genetic modification of the virus to have quicker effect on the vectors	Anderson (1970), Warburg and Pimenta (1995), Jehle et al. (2006), Szewczyk et al. (2009), Szewczyk et al. (2011)
Vital function modification	Sterile Insect Technique (SIT)	Tsetse fly Mosquito	Genetic suppression strategy by creating sterile male vector	Sex segregation of sterile insects in mass production. Inconsistent lifespan affecting release to the wildlife	Alphey and Andreasen (2002), Phuc et al. (2007), and Vreysen et al. (2014)
	Release of Insects carrying a Dominant Lethal (RIDL)	Mosquito	Release of male vector carrying dominant lethal transgene to mate with wild female vector will results in the death of progeny due to the lethal effect from the transgene	Reduced biological fitness of modified insect, affecting release to the wild	Fu et al. (2010), Harris et al. (2012), Carvalho et al. (2015), Dong et al. (2018), and O’Leary and Adelman (2020)
	Genetic Sexing Strain (GSS)	Mosquito New World screwworm fly (Agriculture pest)	Genetically engineered male with insecticide resistance phenotype. Accidentally “leaked” females will be killed by the respective insecticide prior to release	Production difficulty	Fu et al. (2010) and Dong et al. (2018)
	CRISPR/Cas 9 system	Mosquito Sand fly	Manipulation of gene expression to alter vectorial capacity, survival and fertility of vector	Production difficulty, stability issues	Dong et al. (2018)

## Biocontrol *via* predators

The potential of prey–predator relationship in vector control was explored before the era of mass insecticide application. For example, attempts to reduce the larval population of *Stegomyia calopus* (vector of yellow fever) in Ecuador with freshwater fish were initiated as early as the 1910s (Connor, 1922). Various aquatic and amphibian animals were put forward as potential candidates to control mosquito population, based on their predatory nature to the targeted pests. In this review, emphasis is given to medically relevant examples. Of note, most of these predator-driven strategies target the aquatic stages of mosquitoes because the mosquito larvae share a relatively confined living space with the predators. Thus, the aquatic prey–predator encounter does not rely as much on the overlapping active hours of the prey and predator, as compared to the flying adults. In addition, efficient and persistent predation on the vector offspring will inevitably control the vector population, and hence disease transmission (Kumar and Hwang, 2006; Walker and Lynch, 2007; Louca et al., 2009; Griffin and Knight, 2012).

Among the predators, larvivorous fishes have a prolific history as a biocontrol agent against pests, particularly mosquitoes. Larvivorous fishes were introduced into over 60 countries in 20th century to control vector populations (Gerberich and Laird, 1985). Their popularity was attributed mainly to their adaptability to a wide variety of natural and man-made water bodies that serve as mosquito breeding grounds, as well as their rapid reproduction rates (Hadjinicolaou and Betzios, 1973; Motabar, 1978; Chandra et al., 2008a). Numerous field trials with these predators demonstrated between 70 and 97% reduction of mosquito larvae (Connor, 1922; Menon and Rajagopalan, 1978; Fletcher et al., 1992; Kumar et al., 1998; Chandra et al., 2008a; Louca et al., 2009; Griffin and Knight, 2012). For instance, *Aphanius dispar* (Arabian toothcarp) managed to suppress the population of *Anopheles arabiensis* and *Anopheles gambiae* in wells, cisterns and barrels in Djibouti (Louis and Albert, 1988). However, the application of larvivorous fish has raised several concerns. The effect of an alien species to the native fauna and flora needs to be considered and monitored carefully. For example, the continuous introduction of *Gambusia affinis* (Western mosquitofish) into Greece from 1927 to 1937 resulted in the decline of an endemic species *Valencia letourneuxi* (Corfu toothcarp), due to living resource competition between the two species (Economidis, 1995; Economidis et al., 2000). Similar adverse effects associated with *G. affinis* have been reported from Australia and United States (Motabar, 1978; Arthington, 1991; Walton, 2007).

Similarly, odonates (particularly the larvae) are ferocious and imperative predators of many insects. Members of the order Odonata include various dragonflies and damselflies (Shaalan and Canyon, 2009; Vatandoost, 2021). Given their high predation capacity, relatively long aquatic life cycle (usually 1–2 years), and shared aquatic larval habitat with mosquito juveniles, odonates are potential vector biocontrol candidates. Indeed, field trials demonstrated significant reduction of mosquito larvae in water reservoirs by dragonfly nymphs (Sebastian et al., 1980, 1990; Chatterjee et al., 2007; Mandal et al., 2008). For example, a trial release of dragonfly nymphs in Myanmar reported a significant decrease of *Ae. aegypti* population in 2–3 weeks, and the effect persisted till the end of the 4-month-long trial (Sebastian et al., 1990). Similar findings were reported from India (Mandal et al., 2008). The odonate adults are agile aerial predators that prey on many insects (Vatandoost, 2021). Nevertheless, diet analyses of wild-caught dragonfly adults inferred that mosquitoes are rarely taken in large numbers by odonate adults (Pritchard, 1964; Sukhacheva, 1996; Pfitzner et al., 2015). In addition, the active hours (feeding time) of odonate adults (most species are diurnal) do not overlap with the active hours of many medically important vectors (Pfitzner et al., 2015; Vatandoost, 2021). Furthermore, the lifespan of odonate adults is relatively short (1–8 weeks). Hence, the potential of odonate adults as vector biocontrol agents is not as attractive as their juveniles.

The population of many mosquito vectors can be controlled by another mosquito *via* predation. Larvae of mosquitoes from 13 genera prey upon larvae of other arthropods (Harbach, 2007). All members of genera *Toxorhynchites, Lutzia* and *Psorophora* (subgenus *Psorophora*) are obligate predators of other arthropod larvae (Steffan and Evenhuis, 1981; Annis et al., 1990; Rawlins et al., 1991; Collins and Blackwell, 2000; Aditya et al., 2006; Wilkerson et al., 2021; Hancock et al., 2022), whereas larvae of *Sabethes*, several species of *Culex* and *Anopheles* are facultative predators (Lounibos, 1980; Mogi and Chan, 1996; Shaalan and Canyon, 2009; Hancock et al., 2022). Of these, *Toxorhynchites* has received relatively high research attention, mainly because the adult is non-hematophagous (blood feeding), hence not imposing risk as pest or disease vector (Shaalan and Canyon, 2009). Previously, the release of *Toxorhynchites amboinensis* larvae led to a 45% reduction of *Ae. aegypti* population in urban areas of New Orleans (Focks et al., 1985). Similar success was reported with *T. splendens* (Annis et al., 1989; Aditya et al., 2006) and *T. moctezuma* (Rawlins et al., 1991). Apart from their direct effect *via* ferocious predation, the presence of *Toxorhynchites* larvae can delay the prey’s developmental time and increase the prey’s mortality. This is probably due to the stress experienced by the prey in the presence of the predator, or the predator-derived kairomones (Andrade, 2015; Zuharah et al., 2015). Nevertheless, the mechanism behind this effect has yet to be completely deciphered. Despite the earlier reported success, the application of *Toxorhynchites* as biocontrol agent has been hindered by several factors. Firstly, sylvatic species such as *T. rutilus* are not well adapted to urban environment, which restricts its application despite the good predation capacity (Focks et al., 1983). Nevertheless, a more recent surveillance demonstrated the presence of *T. rutilus* in urban areas, albeit of low numbers (Wilke et al., 2019). Indeed, this discovery has reignited the hope of applying *Toxorhynchites* as a vector biocontrol agent in urban areas (Schiller et al., 2019). Besides, the slow population expansion of *Toxorhynchites* is another challenge that needs to be overcome. Under the natural settings, *Toxorhynchites* produces few offspring, which limits their efficacy and capacity in vector biocontrol. This is further aggravated by the cannibalistic nature of *Toxorhynchites* immatures, especially under food-restricted conditions (Donald et al., 2020).

Copepods of genera *Megacyclops, Mesocyclops*, and *Macrocyclops* are crustaceans that feed primarily on the first instar of mosquito larvae. Copepods can adapt to a large variety of water bodies and micro aquatic habitats such as phytotelmata (structures of terrestrial plants that allow formation of water pockets). Such high adaptability allows copepods to be explored as vector biocontrol agents in different settings (Vinogradov et al., 2022). In fact, the discovery of copepod’s potential in vector biocontrol was rather accidental, following an observed reduction of *Ae. aegypti* and *Ae. polynesiensis* larvae from ovitraps set in a study site at Tahiti, after unintentional introduction of copepods to the ovitraps (Riviere and Thirel, 1981). Subsequently, field trials from different regions confirmed the effectiveness of copepods in various water bodies (including drains and land crab burrows) against larvae of medically important mosquitoes, particularly of genera *Aedes* and *Ochlerotatus* (Lardeux et al., 1992; Kay et al., 2002). Importantly, the introduced copepods can adapt and colonize nearby water bodies, allowing sustained effort of mosquito larval control (Kay et al., 2002). Although being used mainly against *Aedes* spp., copepods have been used against other vectors such as *Anopheles* spp. and *Culex* spp. (Riviere and Thirel, 1981; Marten et al., 1989; Lardeux, 1992; Lardeux et al., 1992; Vu et al., 1998; Schaper, 1999; Marten et al., 2000; Kay et al., 2002; Zoppi de Roa et al., 2002; Soumare and Cilek, 2011). Despite their ability to adapt to different sizes of water bodies, copepods are particularly sensitive to temperature changes, chlorine content, low oxygen levels, and presence of toxin within the water (Brown, 1996; Vinogradov et al., 2022). Moreover, it is important to highlight that several species of copepods serve as the intermediate hosts of medically important parasites such as *Drancunculus medinensis* (guinea-worm) and *Dibothriocephalus latus*/ *Diphyllobothrium latum* (fish tape worm; Marten and Reid, 2007; Vinogradov et al., 2022). Therefore, careful consideration and planning must be done prior to application of this method. For example, non-vector copepod species can still be considered as biocontrol agents in certain parts of Africa that are endemic for dracunculiasis (Marten and Reid, 2007).

Water bugs, such as the backswimmers (family: Notonectidae), giant water bugs (family: Belostomatidae) and waterboatmen (family: Corixidae) are important predaceous insects under the order Hemiptera (Shaalan and Canyon, 2009). The potential of *Anisops assimilis* (common backswimmer) to control mosquito population was reported officially for the first time in 1939, following the observation that the backswimmer-harboring water containers were void of mosquito larvae, in contrast to the surrounding backswimmer-free water bodies that were infested with active mosquito larvae (Graham, 1939). Although field and laboratory trials using water bugs to control mosquito larvae exhibited promising results, they are hardly utilized as biocontrol agents due to the high cost and difficulty of mass rearing, as well as logistical challenges (Bay, 1974; Murdock et al., 1984; Venkatesan and Jeyachandra, 1985; Sankaralingam and Venkatesan, 1989; Aditya et al., 2004, 2005, 2006; Selvarajan and Kakkassery, 2019).

Predatory coleopterans from the families Dytiscidae (diving beetle) and Hydrophilidae (water scavenger beetle) are commonly found in ground pools, permanent and temporary ponds (Shaalan and Canyon, 2009). Despite the lower research interest, several studies on the predatory effect of beetles on mosquito reported promising results (Nilsson and Soderstrom, 1988; Juliano and Lawton, 1990; Nilsson and Savensson, 1994; Aditya et al., 2006; Chandra et al., 2008b). However, the efficacy of coleopterans as vector biocontrol agents may be compromised by their diet preference (when mosquitoes are not the only insects presented), species emigration and cannibalism (Juliano and Lawton, 1990; Lundkvist et al., 2003).

Currently, the potential of predators discussed above has not been thoroughly explored, and most of the reported studies focused on mosquitoes (Kim and Merritt, 1987; Werner and Pont, 2003). Notably, several natural predator-based biocontrol strategies have been attempted against non-mosquito vectors, notably the parasitic VBIAR. For example, *Tarentola mautitanica*, an insectivorous lizard, has been proposed as a candidate to control the population of *Triatoma infestans* (kissing bug) that spreads Chagas disease (Castello and Gil Rivas, 1980). Mites and spiders have been suggested as biocontrol agents of *Phlebotomus* spp. (sand fly) that transmits leishmaniasis (Dinesh et al., 2014).

## Pathogenesis-mediated vector biocontrol

Besides predatory animals, pathogens have been proposed as biocontrol agents against vectors. In fact, a number of these pathogens have been applied in the field. These candidates vary in sizes and behavior, encompassing both prokaryotic and eukaryotic organisms. The nematodes are probably the largest candidates on the list. The mermithids are members of an endoparasitic nematode family. These nematodes are highlighted as potential vector biocontrol candidates, due to their parasitic relationship with various arthropods and several arachnids (Stabler, 1952; Chapman, 1974). The hatched pre-parasitic juveniles of mermithid nematodes aggressively infect mosquito larvae (usually the early instars) by paralyzing the targeted hosts, followed by penetration of cuticular wound to establish the infection (Sanad et al., 2017). Once infected, the mermithid parasites take over the cellular function regulatory authority of their hosts. If infection occurs during the early larvae instar, the parasitized mosquito larvae are halted from pupating as the infecting parasites develop within (Stabler, 1952; Allahverdipour et al., 2019). When the nutrient resource supplied by the infected host is exhausted, the nematode, now at its third-stage juvenile post-parasite stage, emerges out of the host, which results in the death of the host (Stabler, 1952). The emerged post-parasite stage then molts into the free-living adult to reproduce and lay eggs. Multiple mermithids may repeatedly infect an already infected larva, giving rise to a phenomenon called superparasitism (Sanad et al., 2017). Different research groups have demonstrated the mosquito larvicidal effect of several mermithids such as *Romanonermis iyengari* (against *Ae. aegypti*, *Ae. albopictus*, *An. gambiae*, *Anopheles culicifacies*, *Anopheles stephensi*, *Anopheles subpictus*, *Armigeres subalbatus*, *Culex pipiens, Culex quinquefasciatus, Culex sitiens, Culex tritaeniorhynchus, and Mansonia annulifera*), *Diximermis peterseni* (against *Anopheles crucians*, *Anopheles quadrimaculatus*, and *Anopheles punctipennis*) and *Strelkovimermis spiculatus* (against *Aedes albifasciatus* and *Cx. pipiens*; Petersen and Willis, 1974; Levy and Miller, 1977; Poinar and Camino, 1986; Santamarina Mijares and Perez Pacheco, 1997; Paily and Balaraman, 2000; Sanad et al., 2013, 2017; Abagli and Alavo, 2019; Abagli et al., 2019). However, the lack of culturable mermithids hinders mass application of this nematode as a biocontrol agent (Kendie, 2020).

Entomopathogenic fungi are another group of insect pathogens that have been explored as a potential vector biocontrol agent (Chapman, 1974). Fungi of genera *Beauveria* and *Metarhizium* have been shown to exert high mortality to medically important mosquitoes of genera *Anopheles*, *Culex* and *Aedes* (Blanford et al., 2011; Accoti et al., 2021). The fungal infection exhausts the mosquitoes due to increased metabolic rate and reduces their frequency of taking blood meals. As a result, the lifespan, oviposition rate, as well as the chance of infected mosquitoes to acquire and transmit medically important pathogens reduces greatly (Blanford et al., 2011). Interestingly, the fungi have been reported to affect both the larval and adult stages of mosquitoes (Blanford, 2005; Blanford et al., 2011). However, the virulence of fungi is influenced by various factors (Scholte et al., 2007; Paula et al., 2011b; Alkhaibari et al., 2017). For example, most fungi may lose their potency after a few months (Scholte et al., 2007). Besides, the lethality of entomopathogenic fungi is influenced by the nutritional state of the targeted vector (Paula et al., 2011b). Furthermore, different forms of fungi may demonstrate different potency against the mosquitoes. For instance, *Ae. aegypti* is more susceptible to the blastospores of *Metarhizium*, whereas *Cx. quinquefasciatus* is more susceptible to the conidia forms. On the other hand, *An. stephensi* is susceptible to both forms of *Metarhizium* (Alkhaibari et al., 2017). Notably, it is difficult to culture and mass produce fungi (Accoti et al., 2021). More importantly, these entomopathogenic fungi have been reported to cause symptomatic infections in immuno-compromised humans, raising safety concerns regarding this vector biocontrol agent (Henke et al., 2002; Tucker et al., 2004; Lara Oya et al., 2016; Goodman et al., 2018). These drawbacks render fungi a less attractive vector biocontrol option.

*Bacillus thuringiensis* var. *israelis* (*Bti*) is a bacterium commonly used as a household larvicide. This bacterium produces delta endotoxins (known as the “Cry” or “Cyt” toxins) during its sporulation, which are potent insecticide proteins (Tabashnik, 1992; Wu et al., 1994; Ben-Dov et al., 1995). The toxin has been demonstrated to kill larvae of *Ae. aegypti* and *Ae. albopictus* effectively, by disrupting the osmotic balance of the midgut epithelial cells upon ingestion (Chapman, 1974; Promdonkoy and Ellar, 2003; Lacey, 2007). Importantly, *Bti* does not pose direct ecological or health threats as it does not affect any off-target organisms including fishes, birds, mammals, and many other insects (Fayolle et al., 2015; Poulin and Lefebvre, 2018; Poulin et al., 2022). Nevertheless, research is underway to evaluate the indirect impact of *Bti* application, particularly its impacts on local ecological systems (Novak et al., 1986; Arredondo-Jimenez et al., 1990; Kumar et al., 1998; Ritchie et al., 2010; Fayolle et al., 2015; Poulin and Lefebvre, 2018; Poulin et al., 2022). Resistance against Cry toxin has yet to be reported. Nevertheless, development of tolerance toward some of the Cry toxins (Cry4Aa and Cry11Aa) was reported in a population of *Ae. sticticus* (Tetreau et al., 2013).

Viruses, such as the mosquito-specific densoviruses (MDV) may be used against the vectors too (Chapman, 1974). MDVs are highly infectious to its targets due to its capability of establishing vertical and horizontal transmission (Johnson and Rasgon, 2018). Upon infection, MDV causes a plethora of pathogeneses on their targets, which lead to apoptosis of infected larvae (Roekring and Smith, 2010), and shortening of adult lifespan (Suchman et al., 2006). Interestingly, MDV has been shown to reduce the viral load of type II DENV in *Ae. albopictus* (Wei et al., 2006). Besides, MDV can be genetically modified to cater for different conditions of vector control. For instance, a recombinant *Ae. aegypti* densovirus (AeDNV) expressing BmK IT1(an insect-specific toxin) was demonstrated to exert higher pathogenicity to *Ae. albopictus* (Gu et al., 2010). Despite these advantages, large-scale implementation of MDV-mediated vector biocontrol strategy may not be easy due to the relatively low stability of viral particles outside the hosts (Johnson and Rasgon, 2018). Nevertheless, advancement of technology may make this method more feasible for mass application in the future.

The potential vector biocontrol candidates above share a drawback that need to be overcome for mass application. It remains uncertain how sustainable these biocontrol agents can exist in the environment for a long-lasting controlling effect against the vector population. This is especially crucial for VBZ and VBIAR with complex and sporadic transmission profiles. Besides, candidates with healthcare risk concerns should not be employed until all doubts are scientifically cleared. Nevertheless, biocontrol candidates such as *Bti* and entomopathogenic fungi have been commercialized recently (Akutse et al., 2020).

## Manipulation of vital biological functions

Alternative approaches that revolve around the manipulation of vector’s biology have been explored to develop a strategy that preserves the relatively target-specific nature of most pathogenesis-mediated biocontrol approaches while overcoming the drawbacks faced by these strategies. Hence, genetic manipulation of vector’s vital functions has gained increasing research attention. Sterile Insect Technique (SIT) is one of the successful examples of such approach (Baumhover et al., 1955; Lofgren et al., 1974; Patterson et al., 1977). In the SIT approach, the male vector is made infertile *via* radiation exposure or chemosterilization (Serebrovsky, 1940; Baxter, 2016). Subsequently, when these sterile males are released into the wild and mate with females, non-viable offspring are produced. As a result, the targeted vector population is reduced. This technique was successfully employed to control the infestation of the New World screwworm fly (*Cochliomyia hominivorax*) in the United States, whose maggots are capable of causing myiasis with severe tissue damages (Baumhover et al., 1955). SIT worked well against *C. hominivorax* because each female fly mates only once. As SIT-modified insects do not produce any offspring, the success of this technique depends on the persistent release of sterile male specimens to compete with the fertile wild type (WT) males for mating. Subsequently, this technique was attempted against mosquitoes in the 1970s, which yielded encouraging results. The population of *Anopheles albimanus* in El Salvador was reduced by 99% after implementing this technique for 5 months (Lofgren et al., 1974). Several mosquito-targeting field trials were performed in Burkina Faso, France, India, Myanmar, and United States. The experimented mosquitoes were *Ae. aegypti*, *An. gambiae, An. quadrimaculatus*, *Cx pipiens*, and *Cx quinquefasciatus* (Weidhaas et al., 1962; Dame et al., 1964; Laven, 1967; Davidson et al., 1970; Patterson et al., 1970; Curtis, 1976; Grover et al., 1976; Patterson et al., 1977; Curtis et al., 1982). These field trials yielded mixed results. For example, in India, the population of targeted mosquitoes was not effectively controlled with this approach, due to the immigration of mated WT females from the locations adjacent to the trial sites. In addition, political turmoil significantly affected the execution of this approach, which confounded the success of this strategy (Curtis, 1976; Curtis et al., 1982).

Despite the reported success, SIT is accompanied with several drawbacks. Firstly, there are concerns among the public members regarding the off-target effect of chemosterilizing agents to the environment (Bartumeus et al., 2019). Laboratory bioassays on non-target predators such as the common house spider (*Achaeranea tepidariorum*) revealed the significant reduction in fertility among the spiders that consumed the chemosterilized mosquitoes (Bracken and Dondale, 1972). Nevertheless, this issue can be overcome *via* simple bulk detoxification using acid and alkaline, which eliminates residues of chemosterilizing agents without compromising the efficacy of this method (Sharma, 1976). Secondly, the difficulty to precisely segregate male and female specimens in the insect colony implies the possibility of sterilizing female specimens by mistake (Sharma et al., 1976; McInnis et al., 1994; Parker, 2005). Accidental release of these mistakenly treated females will result in mating competition with the fertile WT females. As a result, the dispersal of sterile males will be compromised. In addition, radiation used in sterilization will significantly shorten the lifespan of these irradiated insects, which compromises the success of this technique in the field (Alphey and Andreasen, 2002). To overcome this issue, the concept of homozygous female-specific lethal genes has been applied, giving rise to techniques such as Genetic Sexing Strain (GSS) and Release of Insects carrying a Dominant Lethal Gene (RIDL; Franz, 2005; Fu et al., 2010; Harris et al., 2011; Carvalho et al., 2015; O’Leary and Adelman, 2020). RIDL enables selection of the developmental stage corresponding to the manifestation of engineered lethal traits. The insertion of a repressible dominant lethal transgene into the mosquito genome confers conditional fatality (such as tetracycline-dependent survival) to its late juvenile stage. In this approach, the engineered male mosquitoes are released to mate with the WT females. Instead of completing metamorphosis, the produced juveniles that carry a copy of the engineered gene will die in the absence of tetracycline (Phuc et al., 2007). Indeed, field trials of *Ae. aegypti* OX513A in Cayman Islands and Brazil demonstrated strong suppression of the targeted mosquito population (Harris et al., 2012; Carvalho et al., 2015). In addition, female-specific flightless phenotype and DENV-susceptible phenotype that are genetically engineered in *Ae. aegypti* have improved the gender segregation and impeded vector competence to DENV, respectively (de Valdez et al., 2011; Buchman et al., 2020). These techniques minimize the “leakage” of “accidentally treated females” into the wild (Franz, 2002; Calkins and Parker, 2005; Franz, 2005; Koskinioti et al., 2021). In general, the attempts to overcome the shortcomings of SIT revolve around gene editing, which was highly challenging decades ago. However, the discovery and establishment of CRISPR/Cas9 system allows gene editing to be performed much more easily (Gupta et al., 2019). This molecular advancement facilitates the application of SIT against different vectors.

Besides facilitating SIT in vector biocontrol approach, CRISPR/Cas9 can be applied to genetically design arthropod vectors that are not receptive to pathogens transmitted by them under normal circumstances. For example, the knock-out of *FREP1* gene has been shown to reduce the susceptibility of *An. gambiae* to *Plasmodium* spp. (Dong et al., 2018). Gene drive is another genetic engineering concept that has enjoyed a great push in vector control research following the establishment of CRISPR/Cas9 technology. The CRISPR/Cas9- integrated gene drive method allows the targeted genes to be propagated and inherited much more rapidly than the Mendelian rates, resulting in fast replacement or displacement of the targeted traits in a population (Leung et al., 2022). Recently, this technology has been applied on *An. gambiae*, resulting in a successful halting of *Plasmodium* development within the genetically modified mosquitoes, as well as compromising the survival of the homozygous transgenic females (Hoermann et al., 2022). In addition, other gene editing methods, such as the application of homing endonuclease genes (HEG) have been explored to control the malaria vectors (Windbichler et al., 2007; Deredec et al., 2011). Nevertheless, such genetically engineered mosquitoes suffered compromised fitness that hindered their sustainable establishment in the wild. This drawback is in fact a major concern, as modification of one gene may lead to unexpected outcomes on the experimented organism (Resnik, 2014, 2017). If the mutants with unexpected and undesirable traits (following gene editing) thrive in the wild, the ecosystem may be threatened in an unprecedented manner. Nevertheless, the successful application of vital function modification to control a myiasis causative agent with wild and domestic animals as reservoirs reflects the great potential of this approach to control VBZ and VBIAR. Importantly, techniques stemming from this approach should be tested, evaluated, and validated thoroughly before mass application.

## *Wolbachia* as a novel vector biocontrol approach

As elaborated earlier, the pathogenesis-mediated biocontrol agents are arthropod pathogens that shorten the lifespan of vectors, whereas genetic manipulation of arthropod vital functions works by halting the vectors’ population expansion. The application of *Wolbachia* in vector biocontrol is a unique approach that combines the characteristics of both approaches. *Wolbachia* are maternally inherited, gram-negative, obligate intracellular endosymbiotic bacteria found in many arthropods such as mites, spiders, scorpions and isopods (Werren et al., 2008). *Wolbachia* are found in various organs and tissues within the infected arthropod (Werren, 1997; Werren et al., 2008). Besides, medically important filarial nematodes carry *Wolbachia* as well (Lau et al., 2015).

Approximately 60% of the insects are positive for *Wolbachia*. Interestingly, *Ae. aegypti* is *Wolbachia*-free under normal condition (Kittayapong et al., 2000; Rasgon and Scott, 2004). In the early 2000s, Xi et al. (2005) successfully performed an experimental infection on *Ae. aegypti* with *Wolbachia w*AlbB strain (henceforth *w*AlbB) derived from *Ae. albopictus*. Subsequently, this finding was explored further, with trial release of *w*AlbB-infected *Ae. aegypti* in several locations reported increased resistance of the vector to DENV, ZIKV, and CHIKV (Bian et al., 2010; Aliota et al., 2016a,b; Chouin-Carneiro et al., 2019; Nazni et al., 2019). Meanwhile, the infection of *Ae. aegypti* by another more virulent strain of *Wolbachia* (*w*MelPop strain) has been shown to reduce the number of *Ae. aegypti* significantly (Rasgon et al., 2003; Ritchie et al., 2015). These findings highlight the potential of *Wolbachia* as a tool in vector control program. Therefore, the mechanisms behind the effects cast by *Wolbachia* on the infected mosquitoes have received increasing research attention over the past two decades.

*Wolbachia* have evolved and developed various mechanisms to manipulate the host’s cellular biology toward their survival advantage, namely cytoplasmic incompatibility (CI), parthenogenesis, feminization, and male killing (O'Neill et al., 1997; Werren, 1997; Werren et al., 2008). CI happens when a *Wolbachia*-infected male mates with either a *Wolbachia*-negative female or a female infected with a different strain of *Wolbachia*, resulting in non-viable progeny (Werren, 1997). This principle forms the basis of “Incompatible Insect Technique (IIT)” that drives many *Wolbachia*-mediated biocontrol programs against *Ae. aegypti* in China, the United States, and Singapore (Ritchie et al., 2015; Mains et al., 2019; Zheng et al., 2019; Soh et al., 2021). Parthenogenesis refers to the development of eggs into progenies without fertilization, whereas feminization involves development of genetic male into female. *Wolbachia* has been shown to induce feminization in several crustaceans and insects (Werren, 1997; Cordaux et al., 2001; Kageyama et al., 2002; Negri et al., 2006; Werren et al., 2008; Asgharian et al., 2014; Scola et al., 2015). Meanwhile, male killing happens when the affected males experience a significantly shorter lifespan than the affected females. CI, parthenogenesis, feminization, and male killing trigger disruption of gender ratio in the affected population toward female dominance. Using these strategies, *Wolbachia* manipulates the population structure of the infected arthropods, which facilitates the spread and establishment of *Wolbachia* in the wild (Hoffman and Turelli, 1997; Werren, 1997; Werren et al., 2008). Coupled with the reported resistance to virus infection by the *Wolbachia*-infected mosquitoes, the establishment of *Wolbachia* in the vector population may suppress the transmission of these pathogens to humans. In fact, countries such as Malaysia, Indonesia, Laos, Vietnam, Sri Lanka, Australia, Fiji, Vanuatu, Brazil, Colombia, and Mexico have released *Wolbachia* -infected female *Ae. aegypti* to establish a stable *Wolbachia*-infected mosquito colony in the wild (Nazni et al., 2019; World Mosquito Program, 2022). Besides mosquitoes, *Wolbachia* has been explored for the control of black flies and sand flies. However, difficulties in colony maintenance of black flies and sand flies, coupled with the relatively low *Wolbachia* load post-infection in these insects giving rise to the undetectable CI among these insects. This implied the unsuitability of *Wolbachia* for the control of these non-mosquito vectors. Therefore, the versatility of the *Wolbachia* biocontrol approach remains to be validated (Crainey et al., 2010; Bordbar et al., 2014).

Despite the promising advantages of *Wolbachia*-mediated vector biocontrol approach, this method has several shortcomings and concerns. Similar to SIT, the *Wolbachia* method faces the issue of accidental “female leakage” that may compromise the efficacy of IIT-driven vector control strategy. For instance, IIT that incorporates *Wolbachia* can be dampened by mass production. Accidental release of *Wolbachia*-infected females during field trial could affect the population suppression goal. Nevertheless, this may not be considered as an absolute disadvantage, as *Wolbachia*-infected mosquitoes have been claimed to be less susceptible to the medically important pathogens that they carry (Bian et al., 2010; Nazni et al., 2019). To date, the long-term impact of *Wolbachia* on the targeted mosquitoes has not been well studied, partly due to the relatively short discovery history of this vector biocontrol candidate. Furthermore, the interaction dynamics among the arthropod host, *Wolbachia*, and the medically important pathogens carried by the arthropod remains to be fully deciphered. Nevertheless, few studies on this topic revealed interesting findings. For example, a previous study demonstrated that the *Wolbachia*-infected *Culex tarsalis* became more susceptible to West Nile Virus, with much higher viral load post-infection, as compared to the *Wolbachia*-free specimens (Dodson et al., 2014). Since *Wolbachia* has been shown to interfere with interactions between the arthropod host and the medically important pathogens that it carries, it is of utmost importance to consistently assess the efficacy and impact of *Wolbachia* deployed in vector control programs. Besides, the *w*MelPop-related strains have been demonstrated to be temperature-sensitive, raising doubts about the sustainability of this approach in areas with higher temperature (Ulrich et al., 2016; Ross et al., 2017). Succinctly, these concerns deserve more research attention despite the higher research difficulty, where longitudinal study covering adequately long duration is needed.

Besides, concerns have been raised regarding the possibility of *Wolbachia* to cause pathology to humans. Although *Wolbachia* can be found in mosquito salivary glands, the bacteria are not available in saliva, as backed by polymerase chain reaction (PCR) screenings (Wu et al., 2004; Moreira et al., 2009b). In addition, *Wolbachia* are larger than the mosquito salivary duct (Moreira et al., 2009b). Hence, it is relatively unlikely for the bacteria to be transmitted to humans *via* mosquito bites. Moreover, human volunteers exposed to *Wolbachia*-infected mosquitoes over extended period of time revealed absence of antibody specific to *Wolbachia* in their blood (Popovici et al., 2010). Of note, responses to *Wolbachia* or *Wolbachia*-derived antigens by other key players in human immune system remained unclear. Based on currently available information, *Wolbachia* application has been considered as a relatively safe vector biocontrol approach. There are environmental concerns regarding this approach as well. In fact, the major environmental concern is extrapolated from the public health concern, where *Wolbachia* may spread to other organisms across the mosquito-related food chain in the ecosystem. This may disrupt the ecosystem dynamics, hence threatening the biodiversity in the affected environment. Fortunately, *Wolbachia* has been proven to be unable to establish itself throughout the mosquito-associated food chain (Popovici et al., 2010). Notably, natural cross-species infestation of *Wolbachia* is extremely rare (Werren et al., 1995; Werren, 1997), let alone a sustained establishment that allows vertical transmission (Hoffman and Turelli, 1997; Turelli, 2010).

Importantly, *Wolbachia* vector control approach may work well against a disease transmission that involves only one species of arthropod as vector. The effects of *Wolbachia* infection varies with different species of vectors. Hence, infections with multiple vectors, or those with incomplete list of vectors cannot employ this method as vector control program. The clearance of one vector by the bacteria may allow other vectors to thrive, rendering the disease control futile. Knowlesi malaria is an example of VBZ with multiple vectors, and the list of knowlesi malaria vectors is expanding for the moment (Ang et al., 2020; Jeyaprakasam et al., 2020; Pramasivan et al., 2021; Vythilingam et al., 2021). Apart from this, the actual efficacy of *Wolbachia* approach to reduce disease transmission has been questioned. Over the past few years, an increasing number of countries have participated in the release of *Wolbachia*-infected *Ae. aegypti*. Nevertheless, many of these countries still experience increased burden of dengue transmission after persistent release of these mosquitoes (Kementerian Kesihatan Malaysia, 2022). The difficulty to establish stable *Wolbachia* colony within the environment, stability and sustainability of this method in the field, and relative attractiveness of *Wolbachia*-infected mosquitoes during mating may contribute to the challenges faced by this approach to secure a more obvious disease transmission chain breakage in these countries. Obviously, more investigations are needed to better understand this approach, and its practicality, as well as its sustainability in the field.

## Prospects and challenges of vector control against VBZ and VBIAR

**Figure 3:**
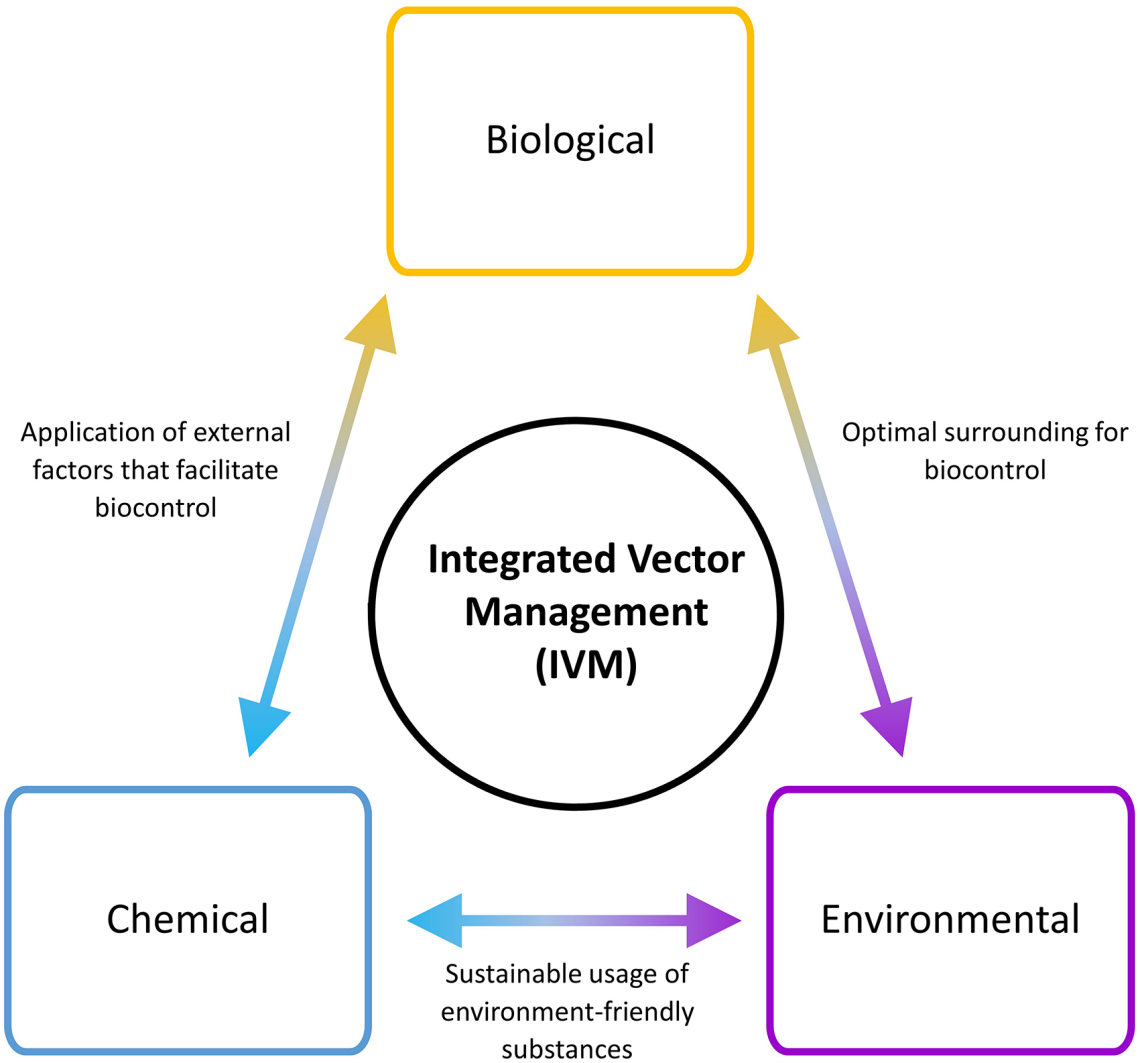
Integrated Vector Management (IVM) involving different components in planning and implementation. Multiple components are factored into an IVM strategy to optimize the output of vector control.

The control and eradication of VBZ and VBIAR hardly rely on a single approach of vector management, due to the complexity of their transmission circuit. Hence, the application of integrated vector management (IVM) that incorporates multiple vector control approaches may increase the success rate of breaking the transmission circuit of these infections (WHO, 2012). To implement a successful and sustainable IVM, the components of IVM triads (biological, environmental, and chemical) should be covered during the designing of the vector control plan (Figure 3). Adaptation and customization of vector control strategies according to the targeted locations are required to ensure high success. For example, the landscape of a targeted location can be modified to facilitate the implementation of vector biocontrol strategies. At the same time, environment-friendly chemicals that can promote biocontrol strategy (such as predator attractants and pheromone-like substances) can be applied. IVM is a multi-prong approach against the vectors, where the selected strategies may complement each other to bring down the vector population. Moreover, IVM may minimize the risk of complete failure faced by a vector control program, as other components in the IVM may continue to work normally when one component is breaking down. For instance, ORS (chemical approach) may be completely stopped during the total lockdown of sudden onset (as exemplified by the COVID-19 pandemic-triggered lockdown in many countries). If the affected area has a well-constructed and maintained drainage system that hampers oviposition by the vectors (environmental management approach), the vector population in that area may not increase after ORS is brought to an abrupt halt. In short, multiple components should be explored to synergize the vector control effort.

While promoting IVM against VBZ and VBIAR, we should not overlook other co-existing factors in the surrounding that may confound the outcomes of the vector control strategy. For example, knowlesi malaria is one of the most prevalent VBZ in Southeast Asia, with Malaysia serving as the epicenter of transmission. Unlike *P. falciparum* that has developed resistance strategies against artemisinin (the current first line anti-malarial treatment; Fairhurst and Dondorp, 2016; Lee et al., 2021), *P. knowlesi* remains susceptible to artemisinin and other anti-malarials in the market (Fatih et al., 2013; van Schalkwyk et al., 2017). Nevertheless, this zoonotic parasite can cause hyperparasitaemia and life-threatening pathogenesis in humans (Lee et al., 2013; Singh and Daneshvar, 2013). Hence, various strategies have been considered to control and eliminate this infection, including the vector management. The potential of different chemical-based approaches has been investigated (Rohani et al., 2020), and IVM against knowlesi malaria transmission has been proposed (Lee et al., 2022). Currently, it is challenging to implement an all-rounded IVM against knowlesi malaria in many hyperendemic areas as the vector profile of this VBZ has yet to be completely deciphered. As mentioned earlier, environment management demands a thorough evaluation of vector profile, transmission dynamics and socio-economic activities in the targeted area. Landscape modification that aims against the incriminated vector may effectively clear the targeted vector’s population. However, the altered landscape may become a conducive breeding ground for another species of anophelines capable of transmitting *P. knowlesi*. Besides, many places affected by knowlesi malaria are endemic to other vector-borne diseases such as dengue and filariasis (Murphy et al., 2020; Zakaria and Avoi, 2022). Therefore, strategies aimed at reducing the transmission of knowlesi malaria should not facilitate the expansion of vectors responsible for other vector-borne diseases. Given the complexity of disease transmission dynamics in many areas endemic to knowlesi malaria, it is not surprising that chemical-based vector control approaches are preferred over other approaches as chemicals are effective against broader range of vectors, despite the potential harmful effects to the environment. Nevertheless, biocontrol strategies with lower target specificity (such as the predator–prey approaches) deserve more attention. Interestingly, edible fishes can be explored as biocontrol candidates, as demonstrated in western Kenya (Howard et al., 2007). The Nile tilapia used in this earlier study is a commonly farmed and eaten fish. In this study, the Nile tilapia significantly reduced the population of *An. gambiae* s.l., *An. funestus* and culicine mosquitoes (Howard et al., 2007). Such integration of vector biocontrol and socioeconomic activity can ensure better sustainability of the implemented vector control efforts. Chemical-based approaches, such as IRS and ORS may be considered and implemented with caution. In addition, environment management *via* human behavioral changes should be emphasized, particularly for VBZ like knowlesi malaria, in which the transmission is associated with socioeconomic activities near or within forested areas, such as tourism, logging, and subsistence cropping (Singh and Daneshvar, 2013; Müller and Schlagenhauf, 2014; Lee et al., 2022). The challenges faced in the control and prevention of knowlesi malaria in Malaysia are applicable to other VBZ and VBIAR. Obviously, there are numerous knowledge gaps that need to be filled with properly designed studies to put forward better vector control programs. Notably, the long-term safety, efficacy, and sustainability of all proposed methods should be investigated thoroughly prior to mass application. Such information is needed to convince the public members and secure their support and compliance to a proposed vector control program, which is crucial to many IVMs. In short, various factors must be taken into consideration when designing a control strategy against VBZ and VBIAR, particularly in areas endemic to multiple vector-borne diseases.

## Conclusion

Vector control has always been a crucial component of breaking the transmission circuit of vector borne diseases. The increased prevalence of vector-borne diseases, including several VBZ and VBIAR in different parts of the world implies a more important role of vector control in healthcare sector. Indeed, there is no “silver bullet” for outbreak management, even more so for the management of the more complex VBZ and VBIAR. Careful integration of multiple vector control approaches in the vector management program may increase the success of disease control and prevention. While battling these pathogens with large investment in the research and development for treatments and vaccines, continuous efforts of discovering novel vector control approaches should be made concurrently, to reduce the prevalence of these infections without compromising the wellbeing of the environment, humans, and the animals involved in the transmission circuit.

## Author contributions

W-CL, MLW, and ZZ conceptualized the review. W-CL, MLW, and ZZ performed literature review and information interpretation. W-CL, MLW, ZZ, IV, YL, MYF, and I-CS involved in manuscript preparation. All authors contributed to the article and approved the submitted version.

## Funding

We would like to thank the Ministry of Higher Education, Malaysia, for the Fundamental Research Grant Scheme (FRGS/1/2022/SKK12/UM/02/9) awarded to W-CL. MLW and MYF were supported by the Long-Term Research Grant Scheme (LRGS) by the Ministry of Higher Education, Malaysia (LRGS/1/2018/UM/01/1/1). IV was supported by another LRGS of the Ministry of Higher Education, Malaysia (LRGS/1/2018/UM/01/1/3). YL was supported by another LRGS of the Ministry of Higher Education, Malaysia (LRGS/1/2018/UM/01/1/4).

## Conflict of interest

The authors declare that the research was conducted in the absence of any commercial or financial relationships that could be construed as a potential conflict of interest.

W-CL is an editorial board member of Frontiers in Microbiology, and one of the guest associate editors for the research topic “Zoonoses – a rising threat to healthcare system.” The authors agree to abide by the rules, ethical standards and practices of Frontiers; and this does not alter the authors’ adherence to all the Frontiers policies on publishing and sharing of information.

## Publisher’s note

All claims expressed in this article are solely those of the authors and do not necessarily represent those of their affiliated organizations, or those of the publisher, the editors and the reviewers. Any product that may be evaluated in this article, or claim that may be made by its manufacturer, is not guaranteed or endorsed by the publisher.
